# Exercise and white matter hyperintensities: a randomized clinical trial

**DOI:** 10.1093/braincomms/fcag381

**Published:** 2026-10-08

**Authors:** Nárlon C Boa Sorte Silva, Roger C Tam, Walid A Alkeridy, Kevin Lam, Elizabeth Dao, Cindy K Barha, Ryan S Falck, Chun Liang Hsu, Ging-Yuek Robin Hsiung, Ken M Madden, Thalia S Field, Teresa Liu-Ambrose

**Affiliations:** Department of Health, Kinesiology, and Applied Physiology, Faculty of Arts and Science, Concordia University, Montréal, Canada H4B 1R6; Department of Physical Therapy, Faculty of Medicine, University of British Columbia, Vancouver, Canada V6T 1Z3; Djavad Mowafaghian Centre for Brain Health, Faculty of Medicine, University of British Columbia, Vancouver, Canada V6T 2B5; Centre for Aging SMART at Vancouver Coastal Health, Vancouver Coastal Health Research Institute, Vancouver, Canada V5Z 1M9; Djavad Mowafaghian Centre for Brain Health, Faculty of Medicine, University of British Columbia, Vancouver, Canada V6T 2B5; Centre for Aging SMART at Vancouver Coastal Health, Vancouver Coastal Health Research Institute, Vancouver, Canada V5Z 1M9; School of Biomedical Engineering, University of British Columbia, Vancouver, Canada V6T 1Z3; Department of Radiology, Faculty of Medicine, University of British Columbia, Vancouver, Canada V6T 1Z3; Department of Medicine, College of Medicine, King Saud University, Riyadh, Saudi Arabia 12372; Division of Geriatric Medicine, Department of Medicine, Faculty of Medicine, University of British Columbia, Vancouver Canada V6T 1Z3; Division of Geriatric Medicine, Department of Medicine, Faculty of Medicine, University of British Columbia, Vancouver Canada V6T 1Z3; Djavad Mowafaghian Centre for Brain Health, Faculty of Medicine, University of British Columbia, Vancouver, Canada V6T 2B5; Department of Radiology, Faculty of Medicine, University of British Columbia, Vancouver, Canada V6T 1Z3; International Collaboration on Repair Discoveries, Blusson Spinal Cord Centre, Vancouver General Hospital, Vancouver Canada V5Z 1M9; Djavad Mowafaghian Centre for Brain Health, Faculty of Medicine, University of British Columbia, Vancouver, Canada V6T 2B5; Hotchkiss Brain Institute, University of Calgary, Calgary, Canada T2N 4N1; Faculty of Kinesiology, University of Calgary, Calgary, Canada T2N 1N4; Department of Physical Therapy, Faculty of Medicine, University of British Columbia, Vancouver, Canada V6T 1Z3; Djavad Mowafaghian Centre for Brain Health, Faculty of Medicine, University of British Columbia, Vancouver, Canada V6T 2B5; Centre for Aging SMART at Vancouver Coastal Health, Vancouver Coastal Health Research Institute, Vancouver, Canada V5Z 1M9; Department of Rehabilitation Sciences, Faculty of Health and Social Sciences, The Hong Kong Polytechnic University, Hung Hom, Kowloon, Hong Kong, China; Djavad Mowafaghian Centre for Brain Health, Faculty of Medicine, University of British Columbia, Vancouver, Canada V6T 2B5; Division of Neurology, Department of Medicine, Faculty of Medicine, University of British Columbia, Vancouver, Canada V6T 1Z3; Centre for Aging SMART at Vancouver Coastal Health, Vancouver Coastal Health Research Institute, Vancouver, Canada V5Z 1M9; Division of Geriatric Medicine, Department of Medicine, Faculty of Medicine, University of British Columbia, Vancouver Canada V6T 1Z3; Djavad Mowafaghian Centre for Brain Health, Faculty of Medicine, University of British Columbia, Vancouver, Canada V6T 2B5; Division of Neurology, Department of Medicine, Faculty of Medicine, University of British Columbia, Vancouver, Canada V6T 1Z3; Vancouver Stroke Program, University of British Columbia, Vancouver, Canada V6T 1Z3; Department of Physical Therapy, Faculty of Medicine, University of British Columbia, Vancouver, Canada V6T 1Z3; Djavad Mowafaghian Centre for Brain Health, Faculty of Medicine, University of British Columbia, Vancouver, Canada V6T 2B5; Centre for Aging SMART at Vancouver Coastal Health, Vancouver Coastal Health Research Institute, Vancouver, Canada V5Z 1M9

**Keywords:** cerebrovascular disease, physical exercise, aging, clinical trial, prevention

## Abstract

Subcortical vascular cognitive impairment (SVCI) is a risk factor for dementia. White matter hyperintensity (WMH) lesions are considered a hallmark of SVCI. We investigated the impact of progressive resistance exercise training (RT) on WMH in individuals with SVCI. This 12-month, single-blinded, proof-of-concept randomized clinical trial involved 89 community-dwelling individuals aged 55 years or older living with SVCI. Participants were randomized (1:1 ratio) to twice-weekly (60 min/day) RT (*n* = 44) or a balance and tone (BAT, *n* = 45) control intervention. Whole-brain and region-specific WMH volumes were measured using T_2_- and proton density-weighted magnetic resonance imaging. Group differences in the 12-month change in WMH from baseline were determined via analysis of covariance, adjusting for baseline WMH, intracranial volume, age, sex and training format (remote versus in-person). Exploratory analyses tested associations between changes in WMH and changes in growth factors. Participants were mostly females (*n* = 58, 65.17%) with an average of 74.50 (SD 5.71) years. There were no differences between groups in whole-brain WMH volume after 12 months [estimated mean difference (95% CI): −0.016 (−0.052 to 0.019) log-transformed cm^3^, *P* = 0.364]. Region-specific analysis showed main effects of training on WMH volume in the temporal lobe [−0.115 (−0.207 to −0.023), *P* = 0.015] and splenium of the corpus callosum [−0.120 (−0.215 to −0.025), *P* = 0.014], favouring the RT group. There was a significant group-by-sex interaction, indicating that intervention effects varied by sex [estimated mean difference-of-differences (95% CI) = −0.093 (−0.170 to −0.016), *P* = 0.019]. *Post hoc* contrasts showed that RT (versus BAT) significantly reduced whole-brain WMH volume in female [−0.049 (−0.093 to −0.005), *P* = 0.029] but not male [0.044 (−0.017 to 0.105), *P* = 0.152] participants. Exploratory analysis showed that increasing levels of vascular endothelial growth factor [standardized *β* (95% CI) = −0.503 (−0.838 to −0.167), *P* = 0.004] were associated with a reduction in whole-brain WMH volume. No associations between insulin-like growth factor 1 and WMH volumes were found (all *P* > 0.05). Based on these findings, we conclude that progressive RT may reduce WMH progression in community-dwelling individuals living with SVCI. This trial is registered with ClinicalTrials.gov, number NCT02669394; submitted for registration on 1 February 2016; the first patient enrolment was on 26 May 2016.

## Teaser

Silva *et al*. report that 12 months of progressive resistance training in adults 55+ resulted in modest reductions in white matter hyperintensity lesion size, a marker of cerebral small vessel disease. The results suggest resistance training may be a valuable non-pharmacological approach to slowing disease progression, particularly among female individuals.

## Graphical Abstract

**Graphical Abstract:**
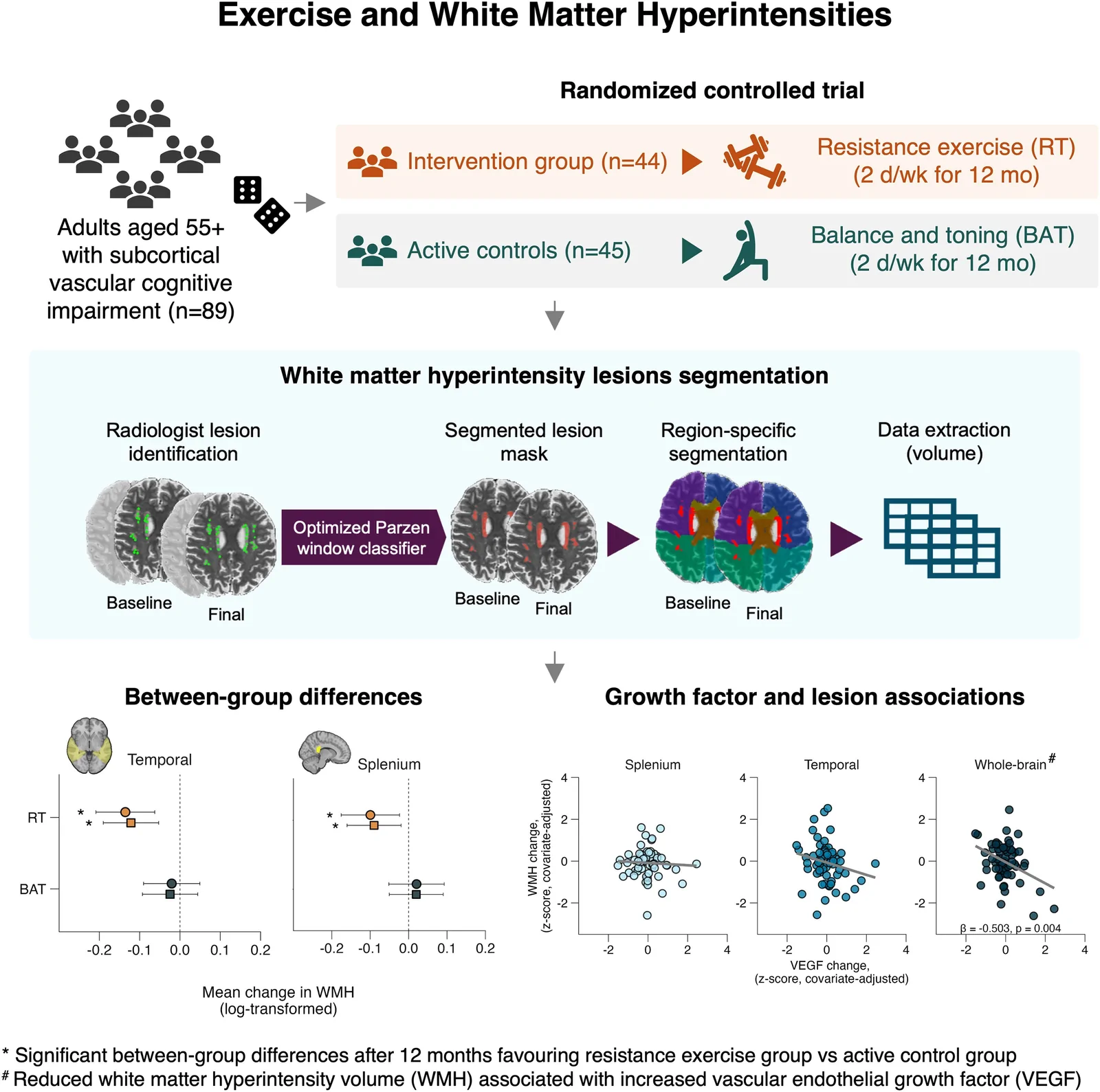

## Introduction

Vascular cognitive impairment is a prevalent syndrome in older individuals.^1^ Subcortical vascular cognitive impairment (SVCI) is its most common form and is typically caused by cerebral small vessel disease.^2^ In SVCI, cerebral small vessel disease commonly manifests as white matter hyperintensity (WMH) lesions on MRI;^3^ WMH are considered a hallmark of SVCI^4^ and disproportionately affect more females than males.^5^

Exercise has well-documented neuroprotective effects^6,7^ and is highly effective in managing cardiometabolic conditions that contribute to the aetiology of SVCI.^8^ Notably, prior studies have shown that resistance training can slow the progression of WMH in older individuals without SVCI.^9,10^ Possible underlying mechanisms include insulin-like growth factor 1 (IGF-1)^11^ and vascular endothelial growth factor (VEGF),^12^ which are involved in the neurobiological adaptations to exercise and may counteract or attenuate SVCI pathology accumulation.^6,7,13-16^

Despite promising evidence, no randomized clinical trials (RCTs) to date have examined the effects of resistance training on WMH progression in individuals living with SVCI. If resistance training can reduce WMH progression in SVCI, it could serve as a widely accessible disease-modifying approach for this population. The objectives of this study were to (1) examine the impact of a 12-month resistance training intervention on WMH progression in individuals living with SVCI, (2) determine the influence of biological sex on intervention effects and (3) evaluate whether changes in exercise-secreted growth factors are associated with WMH changes.

## Materials and methods

### Study design

This was a two-arm parallel, single-blinded, single-site, proof-of-concept RCT conducted in Vancouver, British Columbia, Canada (see Fig. 1). The study protocol has been published.^17^

**Figure 1:**
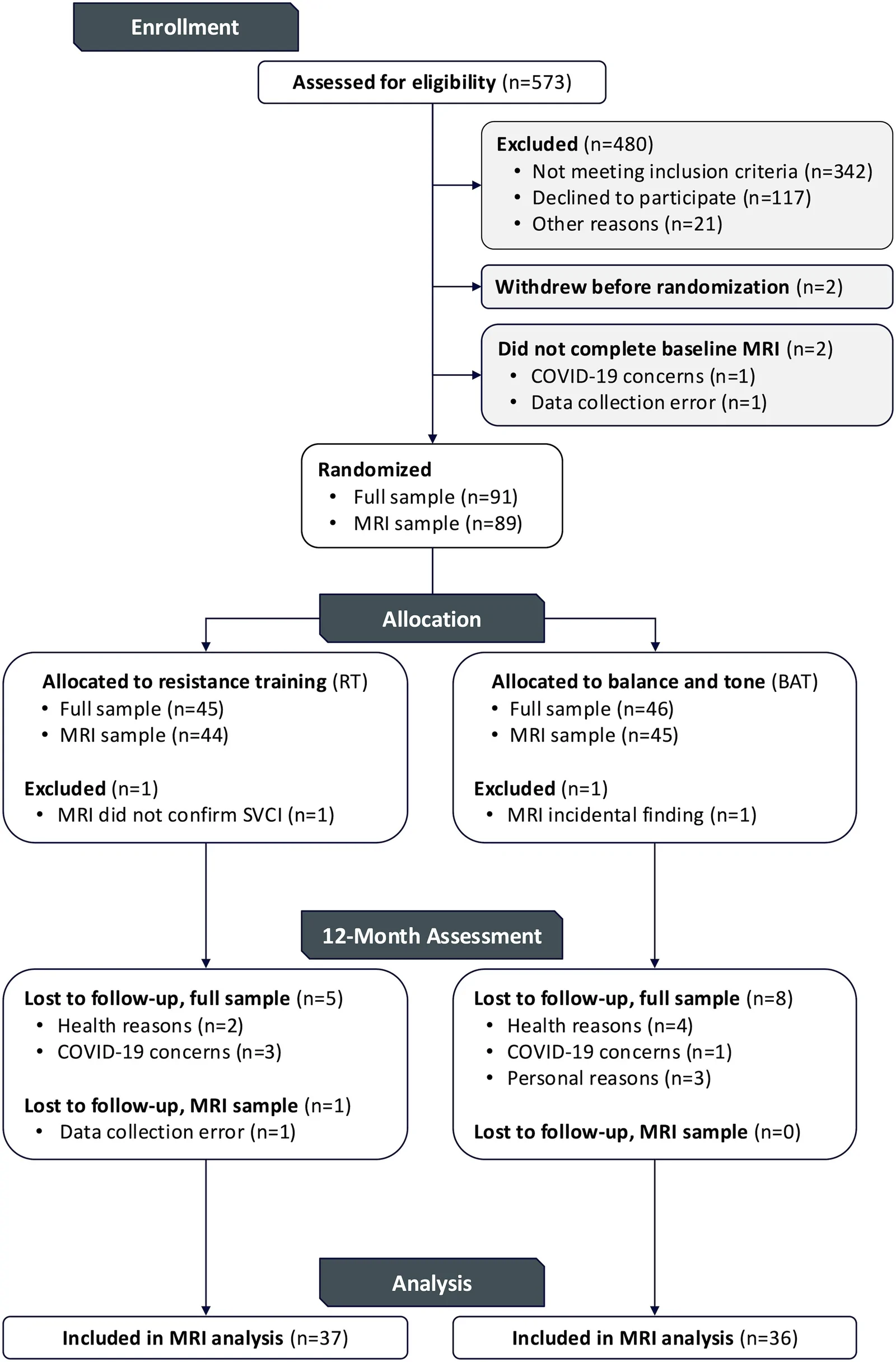
Consolidated standards of reporting trials (CONSORT) diagram showing flow of participants in the study.

### Standard protocol approvals, registrations and patient consents

We received ethical approval from The University of British Columbia Clinical Research Ethics Board (H15-00972) and the Vancouver Coastal Health Research Institute (V15-00972). In accordance with the Declaration of Helsinki, all participants provided written informed consent before study enrolment. There was no involvement of patients or the public in the design, conduct, reporting or dissemination of this research. This study followed the CONSORT reporting guidelines^18^ and was registered on 1 February 2016, with ClinicalTrials.gov (NCT02669394).

### Recruitment

We recruited participants through the Clinic for Alzheimer's Disease and Related Disorders at The University of British Columbia Hospital, through affiliated clinics at Vancouver General Hospital, and via community advertisements.^17^ Enrolment occurred from 26 May 2016 to 17 March 2021.

### Eligibility criteria

Eligible participants were community-dwelling adults who fulfilled the study criteria for SVCI, defined as the presence of mild cognitive impairment^19^ combined with cerebral small vessel disease.^20^ Mild cognitive impairment was operationalized as a Montreal Cognitive Assessment (MoCA) score <26/30 coupled with no clinical diagnosis of dementia, as well as evidence of functional independence, determined through interview and living status (i.e. living independently in one's own home).^17^ Cerebral small vessel disease was assessed based on the presence of WMH (Fazekas Scale ≥1)^17,21^ and/or lacunes on screening computed tomography or MRI. Lacunes were identified following previously established research criteria^17,22^ and were classified dichotomously as present or absent based on visual inspection. Eligibility was confirmed by two trained assessors (W.A.A. and E.D.) who independently reviewed screening scans and baseline study MRI and discussed any discrepancies prior to confirming enrolment. In addition, we used the following inclusion criteria: (1) individuals aged ≥55 years; (2) a Mini-Mental State Examination (MMSE) score ≥20/30; (3) English-speaking; (4) able to comply with scheduled visits, the treatment plan, and other trial procedures; (5) not expected to start or were on a stable fixed dose of cognitive medications during the study; (6) able to walk independently with or without assistive devices; (7) not engaged in resistance training over the last 6 months and (8) provide informed consent.^17^

We excluded participants based on the following criteria: (1) a self-reported diagnosis of neurodegenerative disease, dementia or a genetic cause of SVCI; (2) being at high risk for cardiac complications during exercise; (3) taking medications that may negatively affect cognitive function, such as anticholinergics; (4) planning to participate in, or already enrolled in, a clinical drug trial or exercise trial concurrent with this trial or (5) inability to meet MRI scanning requirements.

### Randomization and blinding

Allocation was concealed. A permuted-block randomization sequence was generated on 26 May 2016 using the website randomization.com (no longer available). The sequence was kept by an investigator who had no role in the day-to-day operations of the trial. Participants were assigned in a 1:1 ratio upon enrolment to one of two 12-month, twice-weekly programmes, which entailed progressive resistance exercise training (RT) or a balance and tone (BAT) active control condition. Assessors were blinded to participants’ allocation, and participants were instructed not to reveal any details of their involvement to assessors. Participants and those who delivered the interventions were not blinded.

### Interventions

Each treatment arm included twice-weekly in-person supervised classes of 60 min for 12 months.^17^ Due to the COVID-19 pandemic, in-person training was not offered from 19 March to 6 August, 2020, 26 November to 8 December, 2020, and 20 April to 29 April, 2021. Instead, participants were asked to train at home. Training sessions were led by certified instructors trained on the study protocol. For at-home training, participants were provided the necessary equipment and access to instructional videos and received monitoring calls weekly.

#### Resistance exercise training

The RT program consisted of exercises using a Keiser^®^ pressurized air system and free weights targeting the upper and lower limbs.^17^ The first four weeks of the program were focused on orienting participants to the exercises and familiarizing them with proper technique. The intensity of training began at a load where participants could complete 2 sets of 10–12 repetitions with proper form. At week 4, training intensity progressed to 70–85% of predicted one-repetition maximum (RM) using the 8RM method; this approach involves identifying the maximum weight a participant can lift for 8 repetitions with correct form, which is then used to estimate their 1RM via a validated prediction equation.^23^ Every 4 weeks, the 8RM test was repeated. For at-home training due to the COVID-19 pandemic, weighted elastic bands were provided.

#### Balance and tone training

The BAT programme consisted of stretching, various core exercises, balance exercises, functional movements, deep breathing and relaxation techniques.^17^ The BAT programme has been used in prior trials of exercise^24-26^ and serves as a control for confounding variables such as physical activity associated with travelling to classes, social interaction and changes in lifestyle secondary to study participation. There is little evidence that BAT exercise improves cognitive health; hence, it was deemed an appropriate active control condition in the present study.^24-26^ Other than body weight, no additional loading was applied to any of the BAT exercises, which were also not progressive in nature, either in loading or difficulty of execution. For at-home training due to the COVID-19 pandemic, BAT participants were provided with a Pilates ball and exercise diaries to document each training session.

### Outcomes

All demographic, clinical and neuroimaging data collection took place at The University of British Columbia and the Vancouver General Hospital within a period of 2 weeks.

#### Demographics and clinical characteristics

Participant demographic baseline data included age, sex, weight, height, body mass index and education. Clinical information was assessed via medical history using the Functional Comorbidity Index.^27^ We assessed global cognitive functioning using the MoCA and the MMSE. Depression symptoms were indexed using the validated 20-item Center for Epidemiologic Studies Depression Scale.^28^

#### White matter hyperintensity volume segmentation

White matter hyperintensity volume was a co-primary outcome in this study. Although study eligibility was determined based on either computed tomography or MRI evidence of cerebral small vessel disease during screening, all participants underwent a standardized study MRI at baseline. All WMH volumes reported in the present analyses were derived from these baseline MRI scans, ensuring consistent lesion quantification across participants regardless of initial screening modality. Detailed imaging parameters can be found in the study protocol.^17^ Briefly, we collected MRI data at The University of British Columbia MRI Research Centre before and after the 12-month intervention. 3D T_2_-weighted and proton density (PD)-weighted scans were used to quantify WMH volume. Anatomical 3D T_1_-weighted scans were also collected and used in preprocessing steps and volumetric analysis.

We quantified WMH volume using an established seed-based, semi-automated segmentation approach.^29^ A trained neurologist (W.A.A.), blinded to group allocation, located WMH lesions and digitally placed seed points on co-registered T_2_- and PD-weighted images. Baseline and follow-up images were longitudinally aligned (baseline as the reference), and seeding was performed simultaneously at both time points by the same rater. These seed points were then fed into a customized Parzen window classifier, which estimated the intensity distribution of the lesions.^29^ Using this method, binary lesion masks were generated and visually inspected to ensure segmentation accuracy. Binary lesion masks derived from this procedure were subsequently reviewed slice-by-slice by a second trained researcher (N.C.B.S.S), also blinded to group allocation, to verify segmentation accuracy.

We further performed region-specific analyses by computing WMH volume in the brain lobes, corpus callosum subregions and deep versus periventricular regions. Following previous methodology,^30,31^ we used the Hammers Adult Atlas^32^ to create binary region masks for the frontal, parietal, temporal and occipital lobes. The corpus callosum subdivisions (splenium, genu and body) were derived using the ICBM-DTI-81 white-matter labels atlas. The lobe-specific and corpus callosum regions were registered to each participant's baseline T_2_-weighted image via non-linear registrations using ANTs (v2.4.2), which allowed quantification of WMH volume for each region separately. Deep and periventricular WMH volumes were computed by segmenting ventricles from T_1_-weighted images using FreeSurfer (v7.1.1). Ventricular binary masks were used to compute distance maps from the ventricular boundary using FSL (v6.0.6), which were thresholded at <8 mm^3^ to capture periventricular lesions or >8 mm^3^ for deep WMH. Data were visually inspected by a trained researcher (N.C.B.S.S.) to ensure accuracy.

Whole-brain and region-specific segmented lesions were quantified as WMH volumes in cm^3^ and log-transformed after adding a constant (0.01 cm^3^) to circumvent the log transformation of zero values.^31^ Additionally, T_2_- and PD-weighted scans were qualitatively assessed using the Fazekas Scale^21^ by trained experts (W.A.A. and E.D.) for descriptive purposes. We also extracted total intracranial volume (ICV) from baseline T_1_-weighted images using FreeSurfer (v7.1.1) to account for differences in brain size in WMH volume in the statistical analysis.^33^

#### Growth factor quantification

Fasting blood was collected via the Clinical Research Unit at Vancouver General Hospital for serum (BD Vacutainer tubes containing clot activator gel, Franklin Lakes, NJ, USA) and plasma (BD Vacutainer tubes containing 10.8 mg of K_2_EDTA). Following blood collection, tubes were inverted five times and held on ice for ∼60 min and then processed. Tubes were centrifuged at 4°C at 2000*×g* for 15 min. Serum and plasma were each aliquoted from the appropriate tubes (clot activator gel or EDTA tubes, respectively) and stored in multiple CryoPure aliquots (Sarstedt, St-Léornard, QC, Canada) at −80°C until further processing for biomarkers.

Growth factor concentrations in serum were measured using two variations of electrochemiluminescence immunoassay kits from Meso Scale Discovery (Rockville, MD, USA). The Custom U-PLEX Metabolic Group 1 (human) Multiplex Assay was used for VEGF A (henceforth referred to as VEGF), and the singleplex R-PLEX Human IGF-1 Assay was used for IGF-1. All samples were thawed on ice and run in duplicate and diluted 1:2 according to the manufacturer's instructions. Plates were read using a Sector Imager 2400 and the Methodical Mind program (Meso Scale Discovery), and data analyses were conducted using Discovery Workbench (v4.0, Meso Scale Discovery). Growth factors had limits of detection ranging from 0.14 to 1560 pg/ml (VEGF) and 107.80 to 40 000 pg/ml (IGF-1). The plate-specific lower limit of detection was defined as the concentration that was 2.5 SD above background. Growth factor measurements with a plate-specific coefficient of variation >15% were excluded from the analysis.

### Sample size calculation

According to preliminary findings,^17^ twice-weekly RT slowed WMH progression in older females with an observed effect size of 0.60. With this effect size, a two-tailed alpha of 0.05, and a beta of 0.20, a sample size of 70 participants (i.e. 35 participants per group) was required to achieve 80% power. We aimed to recruit a total of 88 participants (i.e. 44 participants per group) to accommodate a 20% attrition rate.

### Statistical analysis

We applied analysis of covariance (ANCOVA) to determine between-group differences in whole-brain and region-specific WMH volume changes (12 months minus baseline). The models included baseline WMH volume, ICV, age and sex as covariates. Models were further adjusted for COVID-19-related changes in training format (remote versus in-person). Model estimates and 95% confidence intervals (CI) were extracted using BAT as the reference group.

A pre-specified sex-based analysis^17^ was conducted to determine whether sex moderated the impact of the intervention on whole-brain WMH volume. To assess this, a group-by-sex interaction term was added to the ANCOVA models. The magnitude of the interaction effect was determined based on estimated mean differences and 95% CI for the within-sex, between-group contrast (difference-of-differences). *Post hoc* contrasts were applied to extract sex-specific estimated means and 95% CIs using BAT as the reference category.

To handle missing data, we performed multivariate imputation by chained equations with the R package mice (v3.16.0)^34^ under the assumption that our data were missing at random.^35^ Forty imputed data sets were created with 40 iterations of a Gibbs sampler. A prediction matrix was built to allow baseline and final WMH variables to predict each other. Other predictors included participant demographic and clinical information. We repeated the ANCOVA models after imputation and results were pooled across the imputed data sets.

We conducted exploratory analyses to test the associations between changes in IGF-1 and VEGF with changes in WMH volume using linear regressions, adjusting for ICV, age, sex and training format (remote versus in-person). Extreme outliers exceeding 3 SD above or below the group mean were removed before conducting the regression models.

Lastly, to address potential effects of COVID-19-related protocol changes, we repeated our main analysis of whole-brain WMH volume, stratifying participants by training format (remote versus in-person) to descriptively compare the effects of RT versus BAT across subsamples.

Across all models, we considered two-sided *P* < 0.05 as statistically significant. All statistical analyses were performed in R (v4.4.0) as implemented in R Studio (v2025.09.0+387), using the packages *broom* (v1.0.6), *car* (v3.1.3), *emmeans* (v1.10.2), *patchwork* (v.1.3.2), *performance* (v0.12.0), *psych* (v2.4.3), *tableone* (v0.13.2), and *tidyverse* (v2.0.0).

## Results

### Participant characteristics

The flow of participants during the study is presented in Fig. 1. Measurement of outcomes occurred from 17 May 2016 to 2 June 2022. Mean adherence was 75.1% for the RT group and 70.7% for the BAT group. We had full MRI data available for 87 participants at baseline and 73 at 12 months. Complete-case analysis included all participants (*n* = 73) who completed both the baseline and 12-month assessment (RT, *n* = 37; BAT, *n* = 36) while the analysis of multiple imputed data included all participants (*n* = 87) with baseline MRI data available (RT, *n* = 44; BAT, *n* = 43). Participants were analysed by original assigned groups. Descriptive characteristics of participants are presented in Table 1.

**Table 1:** Demographic and clinical characteristics of study participants

Variables	RT (*n* = 44)	BAT (*n* = 45)
Age, years	74.18 (5.94)	75.00 (5.51)
Sex, *n* (%)		
Female	30 (68.18)	28 (62.22)
Male	14 (31.82)	17 (37.78)
Height, mean (SD), cm	162.43 (9.29)	163.28 (11.11)
Weight, mean (SD), kg	71.82 (17.18)	74.26 (16.22)
Body mass index, mean (SD)^a^	27.12 (5.66)	27.76 (5.28)
Montreal Cognitive Assessment, mean (SD)^b^	21.52 (3.25)	20.89 (3.43)
Mini-Mental State Examination, mean (SD)^c^	27.50 (1.93)	27.13 (1.91)
Center for Epidemiologic Studies Depression Scale^d^	7.02 (5.83)	7.66 (5.93)
Education, *n* (%)		
High school or less	5 (11.36)	8 (17.78)
Trade school or some college	15 (34.09)	10 (22.22)
University degree	24 (54.55)	27 (60.00)
Functional Comorbidity Index, median [IQR]^e^	4.00 [3.00, 4.50]	4.00 [2.00, 5.00]
ICV, mean (SD), cm^3f^	1527.78 (153.27)	1547.47 (173.86)
Fazekas Scale, median [IQR]^g^	1.50 [1.00, 2.00]	1.00 [1.00, 2.00]
Whole-brain WMH, median [IQR], cm^3h^	7.42 [3.97, 12.43]	6.16 [4.26, 15.63]
Lobe-specific WMH, median [IQR], cm^3h^		
Frontal	1.73 [0.74, 3.90]	1.48 [0.71, 5.40]
Temporal	0.53 [0.31, 1.57]	0.47 [0.13, 1.56]
Parietal	0.93 [0.35, 3.55]	0.73 [0.23, 3.52]
Occipital	0.48 [0.18, 0.98]	0.50 [0.18, 1.04]
Corpus callosum WMH, median [IQR], cm^3h^		
Body	0.11 [0.02, 0.33]	0.12 [0.03, 0.33]
Genu	0.27 [0.18, 0.40]	0.31 [0.20, 0.50]
Splenium	0.08 [0.01, 0.21]	0.08 [0.02, 0.23]
Regional WMH, median [IQR], cm^3h^		
Deep	1.75 [0.69, 3.19]	1.66 [0.60, 4.77]
Periventricular	5.32 [2.75, 8.65]	5.00 [3.37, 8.26]
Vascular endothelial growth factor, median [IQR], pg/ml^i^	84.78 [55.94, 133.51]	79.73 [42.18, 153.56]
Insulin-like growth factor, median [IQR], pg/ml^i^	2533.99 [1266.56, 3826.76]	2920.52 [2023.06, 3651.30]

^a^Body mass index is calculated as weight in kilograms divided by height in metres squared.

^b^The Mini-Mental State Examination ranges from 0 (lowest) to 30 (highest); scores 24–30 are the reference range.

^c^The Montreal Cognitive Assessment ranges from 0 (lowest) to 30 (highest); scores 26–30 are the reference range.

^d^Center for Epidemiologic Studies Depression Scale ranges from 0 (lowest) to 60; scores ≤16 are the reference range. Available for 44 in the RT group and 44 in the BAT group.

^e^The Functional Comorbidity Index ranges from 0 (no comorbid illness) to 18 (highest number of comorbid illness). Available for 43 in the RT group and 45 in the BAT group.

^f^Available for 44 in the RT group and 44 in the BAT group.

^g^Fazekas Scale scoring ranges from 0 (lowest) to 3 (highest); higher scores indicate greater white matter lesion burden. Available for 44 in the RT group and 44 in the BAT group.

^h^Available for 44 in the RT group and 43 in the BAT group.

^i^Available for 33 in the RT group and 32 in the BAT group.

BAT, balance and tone group; RT, resistance training group; WMH, white matter hyperintensity.

Overall, the participants were highly educated, mostly female, and presented with several comorbidities. The Fazekas Scale scores indicated that participants had mild to moderate WMH burden.^21^ Of note, there were no differences in whole-brain WMH volume at baseline [*t*(58.6) = 0.43, estimated mean difference = 0.04, 95% CI = −0.14 to 0.22, *P* = 0.667] between male [mean (SD) = 0.83 (0.41), log-transformed cm^3^] and female [0.87 (0.38)] participants or in any of the regional WMH volumes (all *P* > 0.136). Figure 2 shows the spatial distribution of WMH at baseline.

**Figure 2:**
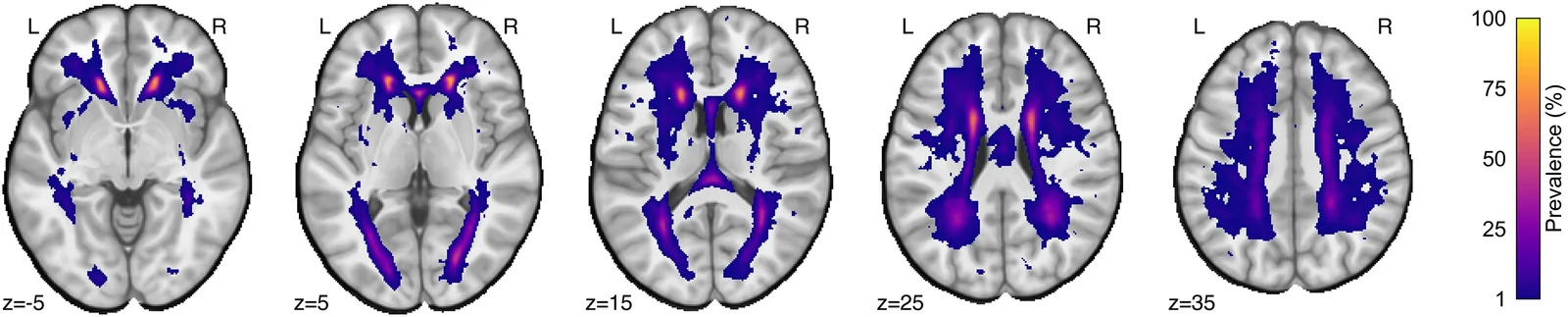
**Prevalence map of white matter lesions at baseline across study participants.** Warmer regions indicate greater prevalence of lesions in the study sample. Data are overlaid on the MNI standard template and were smoothed using a 2 mm full width at half maximum kernel to improve visualization. L, left hemisphere; R, right hemisphere; *z*, axial slice number.

### Protocol changes

The intervention of 21 participants was modified because of the COVID-19 pandemic. Participants were asked to complete at-home training versus in-person training. The 12-month assessment for five participants was delayed by 12 weeks because of the pandemic. During this time, participants continued with at-home training.

### Outcomes

There were no differences between groups in whole-brain WMH volume after 12 months of training (see Table 2). However, we found significant region-specific effects of the intervention wherein RT led to greater reduction in WMH volume in the temporal lobe and splenium of the corpus callosum versus BAT (see Table 2 and Fig. 3). Multiple imputation revealed similar findings for whole-brain and region-specific WMH volume changes (see Table 2 and Fig. 3).

**Figure 3:**
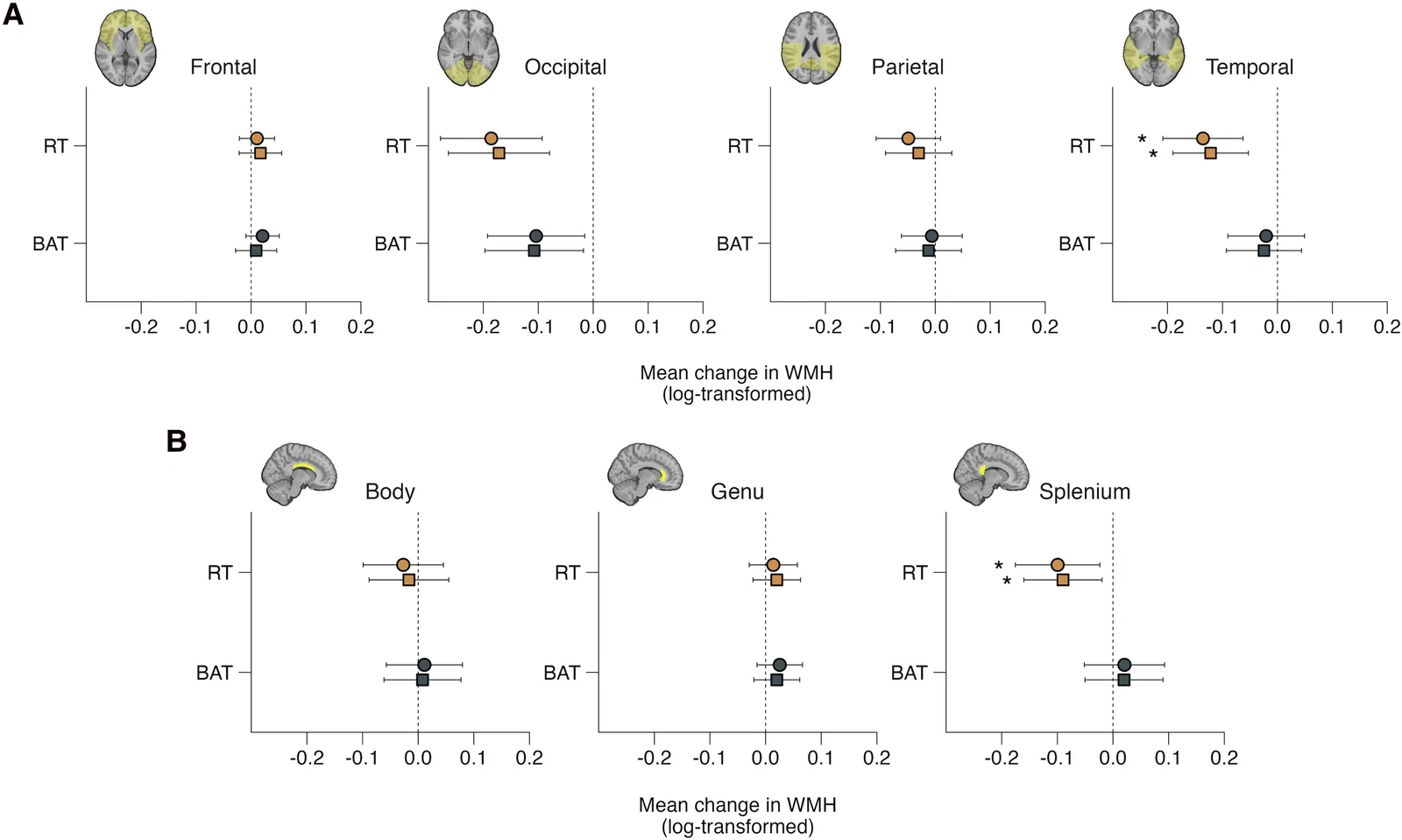
**Region-specific changes in white matter hyperintensity volume after training.** Plots show estimated mean change at 12 months minus baseline for WMH volume in lobar (**A**) and corpus callosum subregions (**B**). Results are point estimates for complete-case (circles) and multiple-imputation (squares) analyses of covariance. Error bars indicate 95% CIs. The WMH volume was log-transformed before analysis. Models were adjusted for baseline WMH volume, ICV, age, sex and training format (remote versus in-person). A total of 73 participants were included in complete-case analysis (RT, *n* = 37; BAT, *n* = 36), and 87 were included in multiple imputed data analysis (RT, *n* = 44; BAT, *n* = 43). *Significant between-group effects at *P* < 0.05 favouring the resistance training group after the intervention. BAT, balance and tone control group; RT, resistance training group; WMH, white matter hyperintensity.

**Table 2:** Estimated mean change and between-group differences at 12 months from baseline on whole-brain and region-specific white matter hyperintensity volume

Outcomes^a^	Estimated mean change (95% CI)^b^		Between-group contrast (95% CI)	*P*-value
	RT	BAT	RT versus BAT	
Whole-brain WMH				
Complete case	−0.022 (−0.051 to 0.007)	−0.005 (−0.033 to 0.022)	−0.016 (−0.052 to 0.019)	0.364
Multiple imputation	−0.010 (−0.043 to 0.023)	−0.007 (−0.040 to 0.025)	−0.003 (−0.044 to 0.039)	0.892
Lobe-specific WMH				
Frontal				
Complete case	0.011 (−0.021 to 0.043)	0.021 (−0.009 to 0.051)	−0.010 (−0.050 to 0.030)	0.614
Multiple imputation	0.017 (−0.022 to 0.056)	0.009 (−0.028 to 0.047)	0.008 (−0.041 to 0.056)	0.748
Temporal				
Complete case	**−0.136 (−0.209 to −0.063)**	**−0.021 (−0.090 to 0.049)**	**−0.115 (−0.207 to −0.023)**	0.015*
Multiple imputation	**−0.122 (−0.190 to −0.053)**	**−0.025 (−0.093 to 0.044)**	**−0.097 (−0.185 to −0.009)**	0.031*
Parietal				
Complete case	−0.049 (−0.108 to 0.010)	−0.006 (−0.062 to 0.049)	−0.043 (−0.116 to 0.030)	0.246
Multiple imputation	−0.030 (−0.091 to 0.030)	−0.012 (−0.072 to 0.048)	−0.018 (−0.092 to 0.056)	0.631
Occipital				
Complete case	−0.186 (−0.278 to −0.093)	−0.104 (−0.192 to –0.015)	−0.082 (−0.197 to 0.034)	0.163
Multiple imputation	−0.171 (−0.264 to −0.079)	−0.107 (−0.197 to −0.018)	−0.064 (−0.176 to 0.048)	0.259
Corpus callosum WMH				
Body				
Complete case	−0.027 (−0.099 to 0.045)	0.011 (−0.057 to 0.080)	−0.038 (−0.128 to 0.052)	0.404
Multiple imputation	−0.017 (−0.088 to 0.055)	0.008 (−0.061 to 0.077)	−0.024 (−0.113 to 0.064)	0.583
Genu				
Complete case	0.014 (−0.029 to 0.057)	0.025 (−0.015 to 0.066)	−0.012 (−0.066 to 0.042)	0.670
Multiple imputation	0.020 (−0.023 to 0.063)	0.020 (−0.021 to 0.061)	0.000 (−0.054 to 0.053)	0.999
Splenium				
Complete case	**−0.100 (−0.175 to −0.024)**	**0.021 (−0.052 to 0.093)**	**−0.120 (−0.215 to −0.025)**	0.014*
Multiple imputation	**−0.090 (−0.160 to −0.020)**	**0.020 (−0.050 to 0.090)**	**−0.110 (−0.202 to −0.018)**	0.020*
Regional WMH				
Deep				
Complete case	−0.064 (−0.114 to −0.013)	−0.021 (−0.069 to 0.027)	−0.043 (−0.106 to 0.021)	0.182
Multiple imputation	−0.048 (−0.101 to 0.005)	−0.019 (−0.069 to 0.031)	−0.029 (−0.095 to 0.037)	0.378
Periventricular				
Complete case	−0.010 (−0.039 to 0.019)	−0.003 (−0.030 to 0.024)	−0.006 (−0.042 to 0.029)	0.723
Multiple imputation	0.000 (−0.033 to 0.034)	−0.010 (−0.043 to 0.022)	0.011 (−0.031 to 0.053)	0.611

^a^Log transformation applied to the white matter hyperintensity volume data. A total of 73 participants included in complete-case analysis (RT, *n* = 37; BAT, *n* = 36), and 87 were included in multiple imputed data analysis (RT, *n* = 44; BAT, *n* = 43).

^b^Change calculated as 12 months minus baseline white matter hyperintensity volume.

*Significant between-group effects (*P* < 0.05) favouring resistance training at 12 months from baseline, adjusting for baseline WMH volume, estimated ICV, age, sex and training format (remote versus in-person).

Bold numbers highlight findings of interest.

BAT, balance and tone control group; RT, resistance training group; WMH, white matter hyperintensity.

We found a significant group-by-sex interaction [*F*(1,65) = 5.82, *P* = 0.019], indicating that the effect of the intervention on WMH volume differed by sex [estimated mean difference-of-differences (95% CI) = −0.093 (−0.170 to −0.016), *P* = 0.019]. *Post hoc* contrasts revealed that RT (versus BAT) significantly decreased whole-brain WMH volume in females but not in males (see Table 3). Multiple-imputation results for the group-by-sex interaction were in the same direction but were not statistically significant (see Table 3).

**Table 3:** Sex-specific estimated mean change and between-group differences at 12 months from baseline on whole-brain white matter hyperintensity volume

Outcomes^a^	Estimated mean change (95% CI)^b^		Between-group contrast (95% CI)	*P*-value
	RT	BAT	RT versus BAT	
Complete case				
Female	**−0.026 (−0.059 to 0.007)**	**0.023 (−0.010 to 0.057)**	**−0.049 (−0.093 to −0.005)**	0.029*
Male	−0.004 (−0.054 to 0.046)	−0.048 (−0.093 to −0.004)	0.044 (−0.017 to 0.105)	0.152
Multiple imputation				
Female	−0.019 (−0.059 to 0.020)	0.009 (−0.032 to 0.050)	−0.028 (−0.081 to 0.025)	0.287
Male	0.006 (−0.052 to 0.064)	−0.037 (−0.090 to 0.016)	0.043 (−0.028 to 0.115)	0.231

^a^Log transformation applied to the white matter hyperintensity volume data. A total of 73 participants included in complete-case analysis (RT, *n* = 37; BAT, *n* = 36), and 87 were included in multiple imputed data analysis (RT, *n* = 44; BAT, *n* = 43).

^b^Change calculated as 12 months minus baseline white matter hyperintensity volume.

*Significant between-group effects (*P* < 0.05) favouring resistance training at 12 months from baseline, adjusting for baseline WMH volume, estimated ICV, age, sex and training format (remote versus in-person).

Bold numbers highlight findings of interest.

BAT, balance and tone control group; RT, resistance training group; WMH, white matter hyperintensity.

Stratified analysis revealed that RT was associated with greater reductions in whole-brain WMH volume compared with BAT among participants who completed remote training [estimated mean difference (95% CI) = −0.080 (−0.140 to −0.020), *P* = 0.013], while results among in-person participants were consistent with the full-sample analysis [−0.005 (−0.050 to 0.041), *P* = 0.842]. These subgroup results should be interpreted with caution given the small remote training subsample (BAT, *n* = 10; RT, *n* = 9).

Analysis of growth factor data showed that increasing levels of VEGF [standardized *β* (95% CI) = −0.503 (−0.838 to −0.167), *P* = 0.004] were associated with a reduction in whole-brain WMH volume. When restricting the analysis to female participants, the association between changes in VEGF and changes in whole-brain WMH volume was attenuated but remained in the same direction [*β* −0.361 (−0.736 to 0.013), *P* = 0.058]. No associations between IGF-1 and WMH volumes were found. Results are presented in Fig. 4 and in Supplementary Table 1.

**Figure 4:**
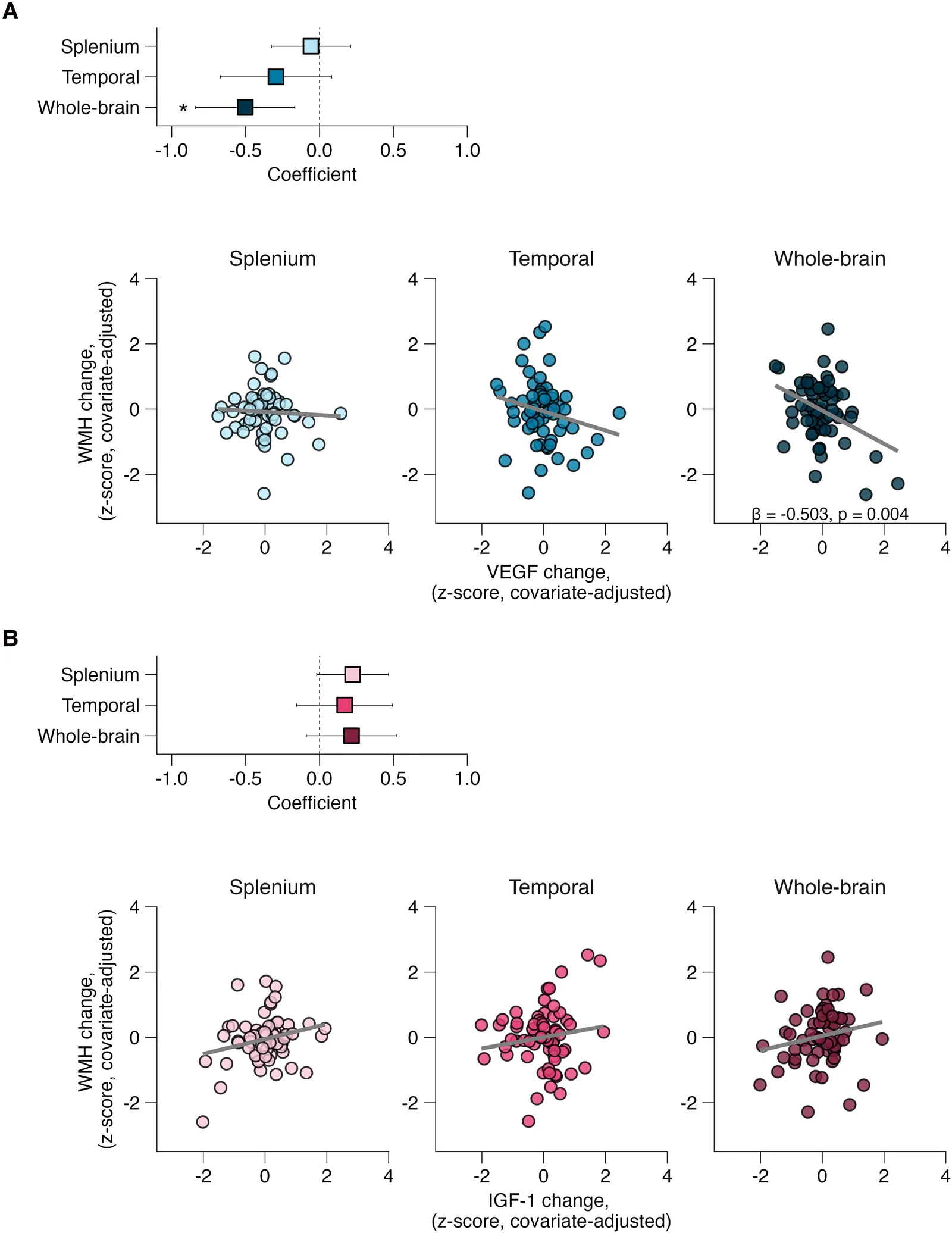
**Associations of change in growth factors and white matter hyperintensity volume.** Panels show results from multivariable linear regression models fitted in complete cases, for VEGF (**A**) and IGF-1 (**B**). In the upper panels, solid squares represent standardized *β* coefficients, and error bars represent the corresponding 95% CIs. All models were adjusted for group, ICV, age, sex and training format (remote versus in-person). In the lower panels, each dot represents an individual participant. Values plotted are residuals from adjusted linear regression models, such that each axis reflects change in WMH volume or change in growth factor after accounting for the covariates listed above. Regression lines correspond to the adjusted model estimates. Only a subsample of participants with growth factor data was analysed in the splenium (*n* = 62), temporal (*n* = 63) and whole-brain (*n* = 62) WMH models. *Significant associations, *P* = 0.004. IGF-1, insulin-like growth factor 1; VEGF, vascular endothelial growth factor A; WMH, white matter hyperintensity.

### Adverse events

A total of 113 adverse events were reported, 63 in the RT group and 50 in the BAT group. Seventy-four events were deemed to be not related or most likely not related to the trial. Of the remaining 39 ‘possibly’, ‘most likely’ or ‘definitely’ related adverse events, 25 were in the RT group and 14 were in the BAT group. No adverse events resulted in study withdrawal or permanent discontinuation.

## Discussion

We investigated the impact of twice-weekly 12 months of a progressive RT programme on WMH volume in individuals aged 55 years and over living with SVCI. We found no main effect of intervention group on whole-brain WMH; however, the region-specific analysis showed a significant effect favouring resistance training compared with the active control condition. Our complete-case analysis further revealed that resistance training reduced WMH progression among females, but not males. We also found that increasing levels of peripheral VEGF over the 12-month study were associated with a reduction in whole-brain WMH volume. Overall, our findings align with the main trial results, in which resistance training was associated with greater improvements in global cognitive function compared with the active control group, with sex-specific effects favouring greater cognitive improvements in female participants.^36^ The present neuroimaging findings, particularly the reduction in WMH volume among female participants,^36^ are consistent with these cognitive outcomes.

Few studies have investigated the effect of exercise on WMH lesion progression.^6^ A 6-month RCT by Suo and colleagues^9^ in individuals living with mild cognitive impairment showed no changes in whole-brain WMH volume after resistance training compared with a sham intervention.^9^ A previous RCT from our group assessing the impact of aerobic exercise in a similar population also revealed no significant effects.^37^ Other RCTs focusing on aerobic exercise^38^ and physical activity counselling^39^ corroborated these null findings. Despite showing no changes in whole-brain WMH volume, the RCT by Suo and colleagues^9^ demonstrated a significant reduction in periventricular and parietal lobe WMH volume.^9^ Our study also showed region-specific effects, namely, WMH volume reduction in the temporal lobe and splenium of the corpus callosum after the 12-month resistance training intervention. The partially incongruent region-specific results between studies may reflect differences in the sample recruited, intervention protocols and WMH quantification and parcellation methods.

It is unclear why resistance training would have such region-specific effects on WMH burden. The current literature postulates that frontal WMH burden is more closely linked to SVCI risk factors, while posterior WMH accumulation appears to be more common in Alzheimer's disease.^31,40-42^ Accordingly, the accumulation of WMH in the temporal lobe and the splenium of the corpus callosum, which are posterior brain regions, has been linked to Alzheimer's disease pathology and neurodegeneration.^40,41^ Although we excluded those with a diagnosis of Alzheimer's disease or any other dementias, it is plausible that our patients also presented with some degree of Alzheimer's disease pathology accumulation, as both SVCI and Alzheimer's disease pathology tend to coexist in most older individuals living with cognitive impairment.^43,44^

Our findings thus pose the question of whether the observed region-specific effects of resistance training on WMH progression can occur via interactions with underlying Alzheimer's disease pathology (e.g. β-amyloid). Exercise mitigates the detrimental effects of β-amyloid on temporal lobe function.^45,46^ These effects are thought to be primarily via irisin,^45,46^ a cytokine secreted by skeletal muscles during exercise.^16^ Muscle-secreted irisin crosses the blood–brain barrier and has neuroprotective effects, including effects on synaptic plasticity and neurogenesis.^7,45,46^ Although its effects are still poorly understood in SVCI, there is some evidence that exercise-induced irisin reduces white matter damage in models of chronic cerebral hypoperfusion,^47^ which is a major factor contributing to SVCI in humans.^48^ Notably, irisin is heavily secreted during resistance training.^49^ This suggests that an exercise increase in irisin is potentially a pathway by which resistance training counteracts WMH progression in regions vulnerable to Alzheimer's disease pathology, such as the temporal lobes and the splenium of the corpus callosum. We did not measure irisin or β-amyloid in our trial, and thus we cannot confirm this hypothesis.

Our complete-case analysis indicated that the intervention effect differed between males and females. *Post hoc* tests further showed that RT reduced WMH volume (versus controls) among female participants.^17^ This apparent sexual dysmorphism in response to exercise may be explained by a greater accumulation and faster progression of WMH burden^5^ coupled with a predisposition to a better vascular response to resistance training in females compared with males.^50,51^ Biological or hormonal differences in response to resistance training^52,53^ may predispose females to experience greater improvements in overall vascular health compared with males.^50,51^ Population-level data indicate that, compared with males, females engage less in muscle-strengthening physical activity but stand to experience a 3-fold greater reduction in cardiovascular disease risk for the same level of engagement.^51^ Greater improvements in cardiovascular health outcomes among females compared with males have been reported in several RCTs.^50,54^ Although these studies did not focus on cerebrovascular health, similar effects could be hypothesized for the brain vasculature, which would result in a greater effect on WMH progression among female participants in response to resistance training. We highlight that sex-specific effects should be interpreted with caution as our multiple-imputation analysis did not corroborate these findings, and the study was not powered to detect sex effects, particularly given the imbalance in sex distribution wherein twice as many females were included across groups.

Changes in VEGF were inversely correlated with changes in whole-brain WMH volume. VEGF is the main growth factor involved in the angiogenic effects of exercise in the periphery of the body and in the brain.^7,13^ Peripherally expressed VEGF plays a key role in preserving end-organ function and extending lifespan in animal models.^55^ Age-related impairment in VEGF signalling leads to critical changes in the microvasculature that compromise blood perfusion and reduce tissue oxygenation, which potentially underlie age-associated pathologies like sarcopenia, osteoporosis and chronic inflammation.^55^ Peripheral VEGF is, therefore, essential to counteracting systemic vascular aging. Although centrally expressed VEGF is the primary driver of angiogenesis and other neurovascular adaptations in the brain,^14^ previous evidence suggests that peripheral VEGF can cross the blood–brain barrier.^15,56,57^ Once in the brain, VEGF can promote cerebral revascularization and stimulate angiogenesis, neurogenesis and neuronal repair.^12,13,15^ Based on this, we postulate that in the context of SVCI, these neurovascular effects of VEGF could translate into a mitigation of WMH burden in response to training.

### Limitations

Our sample comprised highly educated and mostly female participants with low WMH lesion load, which may reduce the generalizability of our findings. We did not collect data on racial and ethnic identities of participants or other measures of socioeconomic status such as income; these data would have provided additional context to the generalizability of our findings. Even though our trial applied well-defined clinical criteria for SVCI, including neuroimaging,^22^ diagnostic frameworks have since evolved,^58^ and our results should be interpreted in this context. Additionally, while our criteria characterized cerebral small vessel disease based on WMH and lacunes, current consensus recognizes a broader spectrum of features (e.g. microbleeds, perivascular spaces and cortical microinfarcts) that were not systematically assessed in our study.^58,59^

Considering the risk of Type I error, the results of analyses investigating region-specific changes in WMH volume should be interpreted with caution as they are exploratory. Before the exploratory analysis, we merged lesions from the right and left hemispheres to mitigate the likelihood of false-positive results. Notably, the COVID-19 pandemic impacted this trial's recruitment, data collection and the delivery of both interventions, which resulted in protocol deviations. Chiefly, the intervention was shifted to a remote format during the shutdown of university research facilities. We attempted to mitigate this by including intervention format (remote versus in-person) as a covariate in our models; however, the overall impact of the pandemic cannot be fully excluded. Of note, our exploratory stratified analysis revealed that among participants who trained remotely, resistance training led to greater reductions in whole-brain WMH volume compared with the active control group. While these findings may reflect intervention-related effects, they should be interpreted with caution given the small sample size and elevated risk of Type I error.

We included only a subsample of participants who had VEGF and IGF-1 data available as blood draws were optional, which may introduce selection bias. We cannot confirm whether the observed associations in our trial were driven primarily by changes induced by resistance training rather than by the active control intervention, nor did we measure central levels of these growth factors to confirm whether peripheral changes reflect changes in the brain. Given that VEGF is the primary growth factor mediating the angiogenic effects of exercise in the brain^7,13^ and that peripheral VEGF can cross the blood–brain barrier to promote cerebral revascularization, angiogenesis and neuronal repair,^12,13,15^ we consider VEGF a mechanistically specific and temporally plausible neurobiological pathway linking resistance training to WMH reduction within the 12-month intervention window of the present study. Nevertheless, other biological pathways may also contribute to the observed effects and cannot be fully excluded. In particular, changes in vascular risk factors, including blood pressure, lipid profile and glycemic status, could represent an indirect route to reduced WMH progression independently of VEGF. Although these factors were monitored during the trial, they were not included in the present analyses, as our study was not designed to formally test their mediating effects. Lastly, the findings from our pre-planned, group-by-sex interaction analysis warrant replication in forthcoming RCTs, as our trial was not originally powered to detect these interaction effects and multiple imputation did not confirm these findings.

## Conclusion

This 12-month trial demonstrated modest but positive effects of progressive RT on regional cerebral small vessel disease burden. Our findings suggest that this type of training may be an important non-pharmacological approach to mitigating disease progression in adults aged 55 and over living with SVCI, especially among female individuals.

## Supplementary Material

fcag381_Supplementary_Data

## Data Availability

Anonymized participant data can be shared upon reasonable request to the corresponding author by a qualified investigator for the purposes of replicating procedures and results. Scripts used to perform region-specific WMH segmentation and to conduct statistical analysis are available in the following GitHub repository: https://github.com/ncbss/exercise-wmh-analysis.
