# Supplementary material for: Exercise and white matter hyperintensities: a randomized clinical trial
Source: Brain Commun. 2026 Oct 8;8(5):fcag381. doi: 10.1093/braincomms/fcag381 (PMC13711657; doi:10.1093/braincomms/fcag381)
Supplement: Supplementary file 1 [file fcag381_supplementary_data.pdf]

Supplementary Material

Exercise and White Matter Hyperintensities: A Randomized Clinical Trial

Nárlon C. Boa Sorte Silva, Roger C. Tam, Walid A. Alkeridy, Kevin Lam, Elizabeth Dao, Cindy K. Barha, Ryan S. Falck, Chun Liang Hsu, Ging-Yuek Robin Hsiung, Ken M. Madden, Thalia S. Field, and Teresa Liu-Ambrose

Supplementary Table 1. Associations between changes in growth factor levels and changes in white matter hyperintensity volumes

Model	Standardized B (95% CI)	t	*P* value	*n*
VEGF				
Splenium	-0.058 (-0.326 to 0.210)	-0.434	0.666	62[a]
Temporal	-0.295 (-0.672 to 0.082)	-1.568	0.122	63[b]
Whole-brain	**-0.503 (-0.838 to -0.167)**	**-3.003**	**0.004***	**62[a]**
IGF-1				
Splenium	0.224 (-0.019 to 0.467)	1.848	0.070	62[a]
Temporal	0.170 (-0.155 to 0.495)	1.049	0.299	63[b]
Whole-brain	0.217 (-0.090 to 0.523)	1.418	0.162	62[a]

[a] Extreme outliers removed before analysis (n=2)
[b] Extreme outliers removed before analysis (n=1)
*Significant associations adjusting for group, estimated intracranial volume, age, sex, and training format (online *vs.* in-person). Log transformation applied to the white matter hyperintensity volume data. All data were standardized before analysis using the z-score transformation.
Abbreviations: IGF-1 = insulin-like growth factor 1; VEGF = vascular endothelial growth factor A
